# Supplementary material for: A Critical View of the Application of the APEX Software (Aqueous Photochemistry of Environmentally-Occurring Xenobiotics) to Predict Photoreaction Kinetics in Surface Freshwaters
Source: Molecules. 2019 Dec 18;25(1):9. doi: 10.3390/molecules25010009 (PMC7017383; doi:10.3390/molecules25010009)
Supplement: Supplementary file 1 [file molecules-25-00009-s001.zip › 3.2.4_gcc-4.4.0.zip!/3.2.4_gcc-4.4.0/3.2.4_gcc-4.4.0/doc/octave/html/interpreter/Comma-Separated-Lists-Generated-from-Cell-Arrays.html]

Comma Separated Lists Generated from Cell Arrays - Untitled


Next: Comma Separated Lists Generated from Structure Arrays,
Up: Comma Separated Lists


---

#### 6.3.1 Comma Separated Lists Generated from Cell Arrays

As has been mentioned above (see Indexing Cell Arrays), elements
of a cell array can be extracted into a comma separated list with the
`{` and `}` operators. By surrounding this list with
`[` and `]`, it can be concatenated into an array. For example:

```
     a = {1, [2, 3], 4, 5, 6};
     b = [a{1:4}]
          => b =
              1   2   3   4
```

Similarly, it is possible to create a new cell array containing cell
elements selected with `{}`. By surrounding the list with
{ and } a new cell array will be created, as the
following example illustrates:

```
     a = {1, rand(2, 2), "three"};
     b = { a{ [1, 3] } }
          => b =
              {
                [1,1] =  1
                [1,2] = three
              }
```

Furthermore, cell elements (accessed by `{}`) can be passed
directly to a function. The list of elements from the cell array will
be passed as an argument list to a given function as if it is called
with the elements as individual arguments. The two calls to
`printf` in the following example are identical but the latter is
simpler and can handle cell arrays of an arbitrary size:

```
     c = {"GNU", "Octave", "is", "Free", "Software"};
     printf ("%s ", c{1}, c{2}, c{3}, c{4}, c{5});
          -| GNU Octave is Free Software
     printf ("%s ", c{:});
          -| GNU Octave is Free Software
```

If used on the left-hand side of an assignment, a comma separated list
generated with `{}` can be assigned to. An example is

```
     in{1} = [10, 20, 30, 40, 50, 60, 70, 80, 90];
     in{2} = inf;
     in{3} = "last";
     in{4} = "first";
     out = cell (4, 1);
     [out{1:3}] = find (in{1 : 3});
     [out{4:6}] = find (in{[1, 2, 4]})
          => out =
             {
               [1,1] = 1
               [2,1] = 9
               [3,1] = 90
               [4,1] = 1
               [3,1] = 1
               [4,1] = 10
             }
```
